# Enhanced parathyroid identification and preservation in total thyroidectomy using combined near-infrared autofluorescence and carbon nanoparticles

**DOI:** 10.3389/fsurg.2026.1793761

**Published:** 2026-06-23

**Authors:** Fan Yu, Zihan Lin, Bo Wu, Quanyong Luo, Jie Kang

**Affiliations:** 1 Department of Nuclear Medicine, Shanghai Sixth People’s Hospital Affiliated to Shanghai Jiao Tong University School of Medicine, Shanghai, China; 2 Department of Thyroid, Breast and Hernia Surgery, Shanghai Sixth People’s Hospital Affiliated to Shanghai Jiao Tong University School of Medicine, Shanghai, China

**Keywords:** carbon nanoparticle, hypoparathyroidism, near-infrared autofluorescence, parathyroid gland, thyroid cancer

## Abstract

**Objective:**

Postoperative hypoparathyroidism remains a common complication after total thyroidectomy. This study aimed to assess whether combining near-infrared autofluorescence (NIRAF) imaging with carbon nanoparticle suspension (CN) improves parathyroid gland (PG) identification and functional preservation in patients undergoing total thyroidectomy for papillary thyroid carcinoma (PTC).

**Methods:**

A total of 80 patients with PTC undergoing total thyroidectomy with central neck dissection (CND) were randomly divided into two groups. The CN group (*n* = 40) received CN alone to distinguish PGs, whereas the NIRAF + CN group (*n* = 40) underwent combined CN and NIRAF imaging for PG identification. Data collected included operative time, counts of identified, incidentally removed, and autotransplanted PGs, and lymph nodes numbers. Serum calcium and parathyroid hormone (PTH) levels were compared preoperatively and postoperatively (on day 1, and at 1, 3, and 6 months) between groups.

**Results:**

The NIRAF + CN group identified significantly more PGs than the CN group (147/160 [91.9%] vs. 135/160 [84.4%], *p* = 0.028), with a lower rate of incidentally removed PGs (5.0% vs. 15.0%, *p* = 0.263) and a higher autotransplantation rate (20.0% vs. 15.0%, *p* = 0.770). On postoperative day 1, serum PTH levels were significantly higher in the NIRAF + CN group (19.86 ± 11.15 pg/mL) than in the CN group (13.82 ± 9.13 pg/mL, *p* = 0.010), while calcium levels and PTH at later time points (1, 3, 6 months) showed no significant intergroup differences. The incidence of transient hypoparathyroidism was 25.0% in the NIRAF + CN group vs. 37.5% in the CN group (*p* = 0.228), and permanent hypoparathyroidism occurred in 2.5% of the NIRAF + CN group compared with 7.5% of the CN group (*p* = 0.615).

**Conclusions:**

The combined use of NIRAF and CN enhances intraoperative PG preservation and improves early parathyroid function.

## 1 Introduction

Total thyroidectomy is the standard surgical treatment for thyroid cancer (1). However, it is frequently complicated by postoperative hypoparathyroidism (2). The parathyroid glands (PGs), essential for calcium homeostasis, are exceptionally vulnerable to inadvertent injury or excision during surgery, leading to postoperative hypocalcemia and hypoparathyroidism. The reported incidence of this complication varies widely, with transient hypoparathyroidism occurring in 5.94%–67.69% and permanent hypoparathyroidism in 0%–20% (3). Therefore, precise intraoperative identification and preservation of PGs is a critical surgical priority.

To address this challenge, innovative tools such as carbon nanoparticle suspension (CN) and near-infrared autofluorescence (NIRAF) imaging have been developed for intraoperative PGs identification. CN is rapidly absorbed by lymphatic vessels (diameter of 20–50 nm) and accumulates in lymph nodes, while PGs are not stained by CN due to their tight capillary endothelium (diameter of 150–500 nm), which prevents CN penetration (4). This creates a negative visual contrast that aids PG identification. Conversely, NIRAF imaging provides a real-time, non-invasive approach for PG identification. It relies on the intrinsic autofluorescence of PG tissue when illuminated by near-infrared (NIR) light (5).

Existing research indicates that NIRAF significantly improves PG identification rates, reducing the incidence of incidental resection and postoperative hypoparathyroidism (6–8). Meanwhile, CN enhances lymph nodes detection during central neck dissection, and aids in PG differentiation and functional preservation (9, 10). Despite these individual benefits, the synergistic potential of combining CN and NIRAF remains under-explored. In this study, we evaluated the efficacy of combing NIRAF with CN techniques, aiming to elucidate the potential benefits in reducing postoperative complications in total thyroidectomy.

## 2 Methods

### 2.1 Study design

A prospective randomized controlled trial was conducted at Shanghai Sixth People's Hospital. A total of 80 consecutive patients underwent total thyroidectomy from April 2024 to September 2024. Patients were randomly assigned to either the CN group or the NIRAF + CN group using sequentially numbered, opaque, sealed envelopes that contained randomized allocation numbers generated by SPSS software. All surgical procedures were performed by a single specialized team with more than 20 years of clinical experience. The study was approved by the Human Subjects Ethics Committee of Shanghai Sixth People's Hospital [Approval Number: 2022-075-(1)]. Written informed consent was obtained from all patients.

### 2.2 Inclusion and exclusion criteria

Inclusion criteria were: (i) age >18 years, (ii) initial total thyroidectomy with unilateral or bilateral central lymph node dissection (CND), (iii) pathologically confirmed diagnosis of papillary thyroid carcinoma (PTC) and (iv) intraoperative use of CN with or without NIRAF imaging.

Exclusion criteria were: (i) advanced thyroid cancer with distant metastasis or major structure invasion, (ii) severe comorbidities contraindicating surgery, (iii) history of prior neck surgery or radiation, and (iv) refusal to participate in the study.

### 2.3 Surgical procedure

All operations were performed by the same surgical team with more than 20 years of experience in thyroid surgery. The standard procedure for all patients consisted of total thyroidectomy with CND. Intraoperative nerve monitoring (IONM) was used to identify and preserve the recurrent laryngeal nerves. Magnifying loupes were not used.

In the CN group, following dissection of the thyroid capsule, 0.1–0.2 mL CN (50 mg, Chongqing Lummy Pharmaceutical Co., Ltd, China) was injected into the thyroid gland using a 1 mL syringe. After 5–10 min of diffusion, the thyroid gland and central lymph nodes were stained black, providing negative contrast to aid in the identification and preservation of unstained PGs. Patients in the CN group underwent surgery without NIRAF imaging assistance.

**Figure 1:**
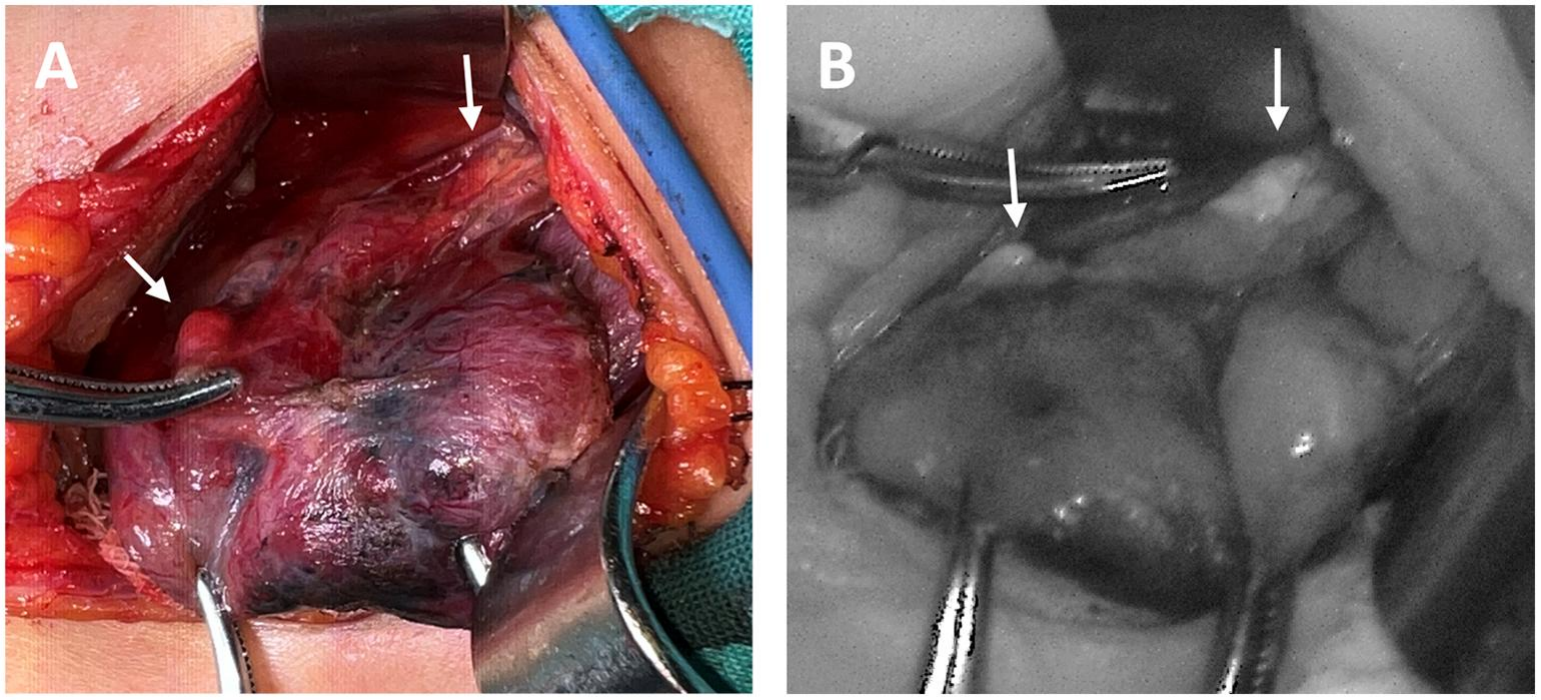
The surgical image **(A)** and corresponding NIRAF image **(B)** of PGs detection in one patient using combined NIRAF and CN approach.

In the NIRAF + CN group, NIRAF imaging was applied after CN injection. A commercially available NIRAF imaging system (ARGOS 300PT, Microscopic Intelligence Co., China) was utilized for intraoperative PGs identification. The system emits light at 785 nm to induce tissue autofluorescence, which is detected at 820 nm. For optimal imaging, the camera was positioned at 15 cm above the surgical field, with the NIR light perpendicular to the tissue surface. The ambient light was turned off to minimize interference from external light sources when detecting PGs. A single trained assistant operated the system and adjusted the camera position as needed to optimize visualization. NIRAF imaging was systematically performed at three stages: Stage I (after dissection of the thyroid capsule), Stage II (after thyroid gland removal), and Stage III (on the removed specimens) (11). Suspected PGs identified intraoperatively were validated using the immune colloidal gold technique (ICGT), a rapid method with diagnostic accuracy comparable to frozen section analysis (12). The visualization results of NIRAF combined with CN are shown in Figure 1.

### 2.4 Observations

The number of identified PGs, incidence rate of incidentally removed PGs, and the PGs autotransplantation rate were recorded. The parathyroid function was evaluated by measuring serum parathyroid hormone (PTH) and calcium levels preoperatively and postoperatively (on day 1, and at 1, 3, and 6 months), with concurrent documentation of postoperative hypocalcemia and rates of transient and permanent hypoparathyroidism. Hypocalcemia was defined as a serum calcium level below 2.10 mmol/L. Transient hypoparathyroidism was defined as meeting the diagnostic criteria for hypoparathyroidism (PTH <15 pg/mL, or PTH ≥15 pg/mL with hypocalcemia requiring calcium supplementation) within 6 months postoperatively, with complete resolution by 6 months. Permanent hypoparathyroidism was defined as meeting the same diagnostic criteria (PTH <15 pg/mL, or PTH ≥15 pg/mL with hypocalcemia requiring calcium supplementation) beyond 6 months postoperatively (13). In addition, baseline characteristics included gender, age, days of hospitalization, operation time, surgical approach, tumor size, total number of central lymph nodes dissected, and number of central lymph nodes with metastasis were recorded.

### 2.5 Statistical analysis

Statistical analysis was performed using SPSS software (version 26.0). Data normality was assessed using the Kolmogorov–Smirnov test. Categorical variables were compared using the chi-square test or Fisher's exact test, while continuous variables were analyzed using the independent t-test or Mann–Whitney *U* test. Continuous variables were presented as mean ± standard deviation (SD), while categorical variables were presented as frequencies and percentages. *P* < 0.05 was considered statistically significant.

## 3 Results

### 3.1 Baseline clinical characteristics

A total of 80 patients who underwent total thyroidectomy were included in this study. The detailed clinical characteristics of the two groups are presented in Table 1. No statistically significant differences were observed between the NIRAF + CN group and the CN group regarding gender (*p* = 0.790), age (*p* = 0.842), days of hospitalization (*p* = 0.263), operation time (*p* = 0.692), tumor size (*p* = 0.809), tumor side (*p* = 0.715), tumor position (*p* = 0.765), presence of Hashimoto's thyroiditis (*p* = 0.612), and surgical procedure (*p* = 0.823).

**Table 1:** Clinical characteristics of 80 patients in the study.

Characteristics	NIRAF + CN group (*n* = 40)	CN group (*n* = 40)	*P* value
Gender, *n* (%)			
Male	10 (25.0%)	8 (20.0%)	0.790
Female	30 (75.0%)	32 (80.0%)	
Age (years, Mean ± SD)	48.03 ± 14.62	48.67 ± 12.41	0.842
Days of hospitalization (days, Mean ± SD)	5.42 ± 1.27	5.83 ± 1.81	0.263
Operation time (minutes, Mean ± SD)	96.67 ± 20.81	99.03 ± 28.85	0.692
Tumor size (mm, Mean ± SD)	9.50 ± 7.25	9.43 ± 8.66	0.809
Tumor side^a^, *n* (%)			
Left	30 (50.0%)	27 (45.0%)	0.715
Right	30 (50.0%)	33 (55.0%)	
Hashimoto's thyroiditis, *n* (%)			
Yes	12 (30.0%)	9 (22.5%)	0.612
No	28 (70.0%)	31 (77.5%)	
Tumor position^a^, *n* (%)			
Superior pole	22 (34.4%)	17 (28.3%)	0.765
Middle pole	22 (34.4%)	20 (33.3%)	
Anterior pole	20 (31.2%)	23 (38.3%)	
Procedure, *n* (%)			
Total thyroidectomy with unilateral CND	18 (45.0%)	20 (50.0%)	0.823
Total thyroidectomy with bilateral CND	22 (55.0%)	20 (50.0%)	

a Tumor side and tumor position are presented by number of lesions.

### 3.2 Intraoperative findings and postoperative complications

A total of 147 PGs were intraoperatively identified in the NIRAF + CN group, compared with 135 in the CN group. When normalized to per patient, the mean number of identified PGs was significantly higher in the NIRAF + CN group than in the CN group (*p* = 0.028). The incidence of incidentally removed PGs was lower in the NIRAF + CN group (5.0%, 2/40) than in the CN group (15.0%, 6/40), though this difference did not reach statistical significance (*p* = 0.263). Additionally, the PGs autotransplantation rate was higher in the NIRAF + CN group (20.0%, 8/40) compared with the CN group (15.0%, 6/40), with no statistically significant difference (*p* = 0.770). Regarding lymph node dissection outcomes, no significant differences were observed between the two groups in the number of central lymph nodes dissected (*p* = 0.971) or the number of metastatic central lymph nodes (*p* = 0.600).

For postoperative complications, the incidence of vocal cord paralysis was 2.5% (1/40) in the NIRAF + CN group and 5.0% (2/40) in the CN group (*p* = 1.000). On postoperative day 1, the incidence of hypocalcemia was lower in the NIRAF + CN group (10.0%, 4/40) than in the CN group (20.0%, 8/40), without statistical significance (*p* = 0.348). The intraoperative findings and postoperative complications are detailed in Table 2.

**Table 2:** Intraoperative findings and postoperative complications.

Outcome	NIRAF + CN group	CN group	*P* value
Number of PGs identified (*n*, Mean ± SD)	3.67 ± 0.57	3.38 ± 0.63	0.028
Incidentally removed PGs, *n* (%)	5.0% (2/40)	15.0% (6/40)	0.263
PGs autotransplantation, *n* (%)	20.0% (8/40)	15.0% (6/40)	0.770
Central lymph node dissected (*n*, Mean ± SD)	5.48 ± 3.19	5.55 ± 2.97	0.971
Central lymph node metastases (*n*, Mean ± SD)	2.33 ± 1.94	2.08 ± 1.80	0.600
Vocal cord paralysis, *n* (%)	2.5% (1/40)	5.0% (2/40)	1.000
Hypocalcemia, *n* (%)	10.0% (4/40)	20.0% (8/40)	0.348
Hypoparathyroidism, *n* (%)			
Transient	25.0% (10/40)	37.5% (15/40)	0.228
Permanent	2.5% (1/40)	7.5% (3/40)	0.615

### 3.3 Parathyroid function outcomes

**Figure 2:**
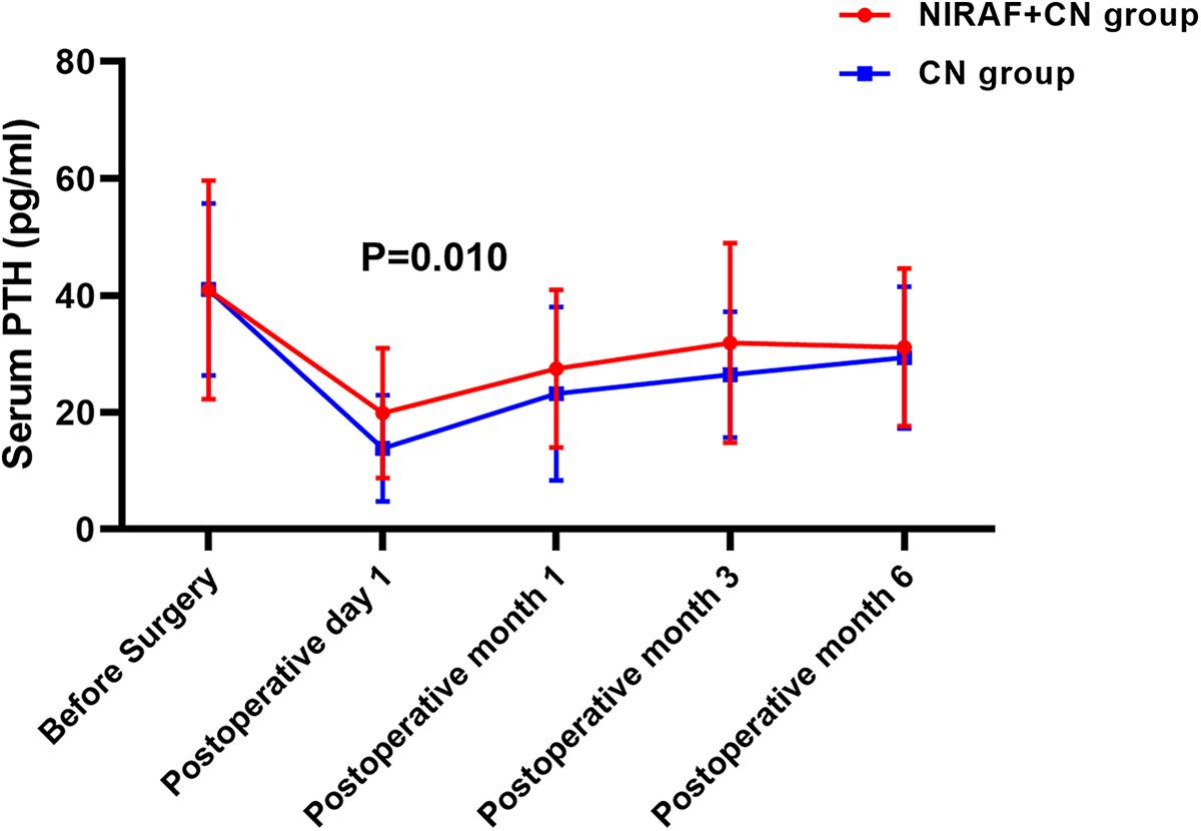
Changes in serum PTH in the two groups during the 6-month postoperative period.

**Figure 3:**
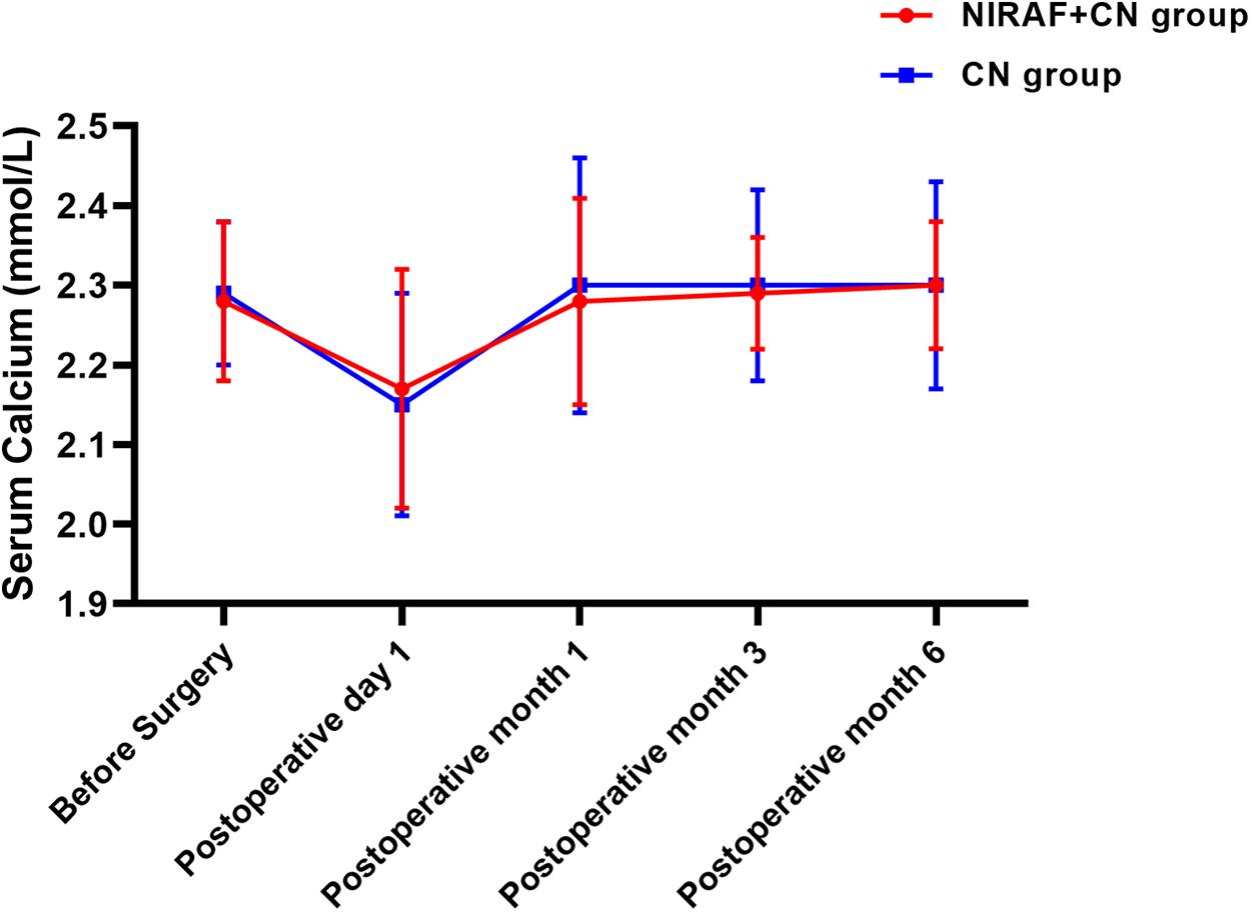
Changes in serum calcium in the two groups during the 6-month postoperative period.

Serum PTH and calcium levels are presented in Table 3 and illustrated in Figures 2, 3. Preoperative PTH and calcium levels were comparable between groups. On postoperative day 1, serum PTH levels were significantly higher in the NIRAF + CN group (19.86 ± 11.15 pg/mL) compared to the CN group (13.82 ± 9.13 pg/mL, *p* = 0.010). Serum calcium levels on day 1 were not significantly different (*p* = 0.431). No significant differences in serum PTH or calcium levels were observed at the 1-month, 3-month, and 6-month follow-up time points. The incidence of transient hypoparathyroidism was 25.0% (10/40) in the NIRAF + CN group and 37.5% (15/40) in the CN group (*p* = 0.228). Permanent hypoparathyroidism occurred in 2.5% (1/40) of patients in the NIRAF + CN group and 7.5% (3/40) in the CN group, with no significant differences (*p* = 0.615).

**Table 3:** Intraoperative findings and postoperative complications^a^.

Variables	NIRAF + CN group	CN group	*P* value
Serum PTH (pg/mL, Mean ± SD)			
Before surgery	40.96 ± 18.74	40.99 ± 14.71	0.624
Postoperative day 1	19.86 ± 11.15	13.82 ± 9.13	0.010
Postoperative month 1	27.45 ± 13.49	23.20 ± 14.81	0.088
Postoperative month 3	31.90 ± 17.07	26.47 ± 10.81	0.285
Postoperative month 6	31.14 ± 13.51	29.34 ± 12.18	0.534
Serum calcium (mmol/L, Mean ± SD)			
Before surgery	2.28 ± 0.10	2.29 ± 0.09	0.781
Postoperative day 1	2.17 ± 0.15	2.15 ± 0.14	0.431
Postoperative month 1	2.28 ± 0.13	2.30 ± 0.16	0.605
Postoperative month 3	2.29 ± 0.07	2.30 ± 0.12	0.868
Postoperative month 6	2.30 ± 0.08	2.30 ± 0.13	0.941

a Reference ranges: Serum calcium, 2.10–2.55 mmol/L; PTH, 15–65 pg/mL.

## 4 Discussion

Postoperative hypoparathyroidism remains the most frequent complication following thyroid surgery, particularly total thyroidectomy with CND. This is primarily due to PG anatomical variability and vascular vulnerability during surgery (14). Several studies reported that the incidence of transient hypoparathyroidism after total thyroidectomy with CND for differentiated thyroid carcinoma ranges from 17.8% to 48.69% (15–17). Therefore, accurate intraoperative identification and preservation of functional PGs have become a primary surgical focus for surgeons.

To enhance intraoperative PGs identification, the CN technique is widely used in traditional and endoscopic thyroidectomy, because of its rapid staining, long duration and clear lymphatic tracing (14). Lin et al. (18) compared conventional surgery with CN-assisted surgery in bilateral thyroidectomy with CND and found that CN use was associated with better postoperative parathyroid function, as evidenced by more stable serum calcium levels, higher PTH levels, and a significantly lower rate of PG injury. Meanwhile, NIRAF imaging is increasingly recognized as an effective approach for intraoperative PGs identification. Paras et al. first demonstrated that PGs exhibit autofluorescence when exposed to 785 nm NIR light (19). In our previous study, NIRAF demonstrated a sensitivity of 95.4%, a specificity of 77.5%, a positive predictive value of 90.5%, and a negative predictive value of 88.1% (11). When used alone in total thyroidectomy with CND, each technique has inherent limitations: CN can fail to detect small or ectopic PGs, while NIRAF is prone to false-positive and false-negative readings due to interference from surrounding adipose tissue or blood, respectively (20).

Therefore, we further combined these two techniques to observe whether they could improve the parathyroid function in patients. In our work, we evaluated the effectiveness of combined NIRAF and CN techniques in patients undergoing total thyroidectomy with CND. The NIRAF + CN group identified significantly more PGs than the CN group (147/160 [91.9%] vs. 135/160 [84.4%], *p* = 0.028). A higher number of incidentally removed PGs was confirmed by pathological examination in the CN group (6 cases) compared to the combined group (2 cases). Meanwhile, the autotransplantation rate of PGs was 20.0% (8/40) in the NIRAF + CN group and 15.0% (6/40) in the CN group, respectively. These results indicated that the combined application of NIRAF and CN enhanced intraoperative PGs protection, minimizing the risk of inadvertent resection.

Serum PTH and calcium levels were systematically compared between the two groups. On postoperative day 1, PTH levels in the NIRAF + CN group were significantly higher than those in the CN group (*p* = 0.010), indicating better-preserved parathyroid function in the immediate postoperative period. At subsequent time points up to six months, no statistically significant differences in PTH or calcium levels were observed between the groups. These findings suggest that the combined application of NIRAF and CN primarily mitigates early postoperative parathyroid dysfunction, which is the critical period for hypocalcemia-related morbidity. We hypothesize that this transient protective effect may be attributable to the enhanced intraoperative identification and avoidance of inadvertent injury to the *in-situ* PGs facilitated by NIRAF imaging, thereby preserving function during the acute phase of surgical recovery.

Complete lymph node dissection is equally critical for patients with PTC compared to PG preservation. In our study, there were no significant differences observed in either the total number of central lymph nodes dissected (*p* = 0.971) or the number of metastatic lymph nodes (*p* = 0.600) in two groups. This suggests that the addition of NIRAF imaging does not augment the efficacy of lymph nodes dissection, and the primary role of CN in lymphatic mapping remains central to achieving thorough nodal clearance. Therefore, the benefit of combining NIRAF with CN appears to be largely confined to enhancing PGs identification and preservation.

To our knowledge, this is the first study to compare the efficacy of the combine technique (NIRAF + CN) with that of CN technique in total thyroidectomy with CND, investigating the value of the integrated approach. However, our study has several limitations. First, the sample size of the study was small, which may restrict its external validity and statistical power. Consequently, the study may have been underpowered to detect statistically significant differences in secondary outcomes such as hypoparathyroidism rates. Second, patients were enrolled in a single center, which may have led to a selection bias. Third, the follow-up period of six months is adequate for assessing permanent hypoparathyroidism, but longer-term data would provide a more complete outcome of parathyroid function. Further research should focus on validating these promising results through larger, multi-center, prospective studies with extended follow-up to conclusively define the role of combined NIRAF and CN in standard surgical practice and its long-term impact on patient outcomes.

## 5 Conclusion

The combined application of NIRAF imaging and CN significantly enhances intraoperative PGs identification and early postoperative parathyroid function compared to CN alone during total thyroidectomy with CND. The integration of the two methods is a valuable adjunct to established techniques to improve perioperative safety and optimize patient outcomes.

## Data Availability

The raw data supporting the conclusions of this article will be made available by the authors, without undue reservation.
